# Clinical potential of gene mutations in lung cancer

**DOI:** 10.1186/s40169-015-0074-1

**Published:** 2015-11-24

**Authors:** Miranda B. Carper, Pier Paolo Claudio

**Affiliations:** Lineberger Comprehensive Cancer Center, University of North Carolina at Chapel Hill, Chapel Hill, NC 27599 USA; Department of Radiation Oncology, The University of Mississippi Medical Center Cancer Institute, 350 W Woodrow Wilson Ave, Jackson, MS 39213 USA; Department of Biomedical Sciences, University of Mississippi, National Center for Natural Products Research, Oxford, MS USA

**Keywords:** NSCLC, Mutations, Targeted therapy, Personalized therapy, Biomarkers, Bench-to-bedside

## Abstract

Lung cancer is the most common cancer type worldwide and the leading cause of cancer related deaths in the United States. The majority of newly diagnosed patients present with late stage metastatic lung cancer that is inoperable and resistant to therapies. High-throughput genomic technologies have made the identification of genetic mutations that promote lung cancer progression possible. Identification of the mutations that drive lung cancer provided new targets for non-small cell lung cancer (NSCLC) treatment and led to the development of targeted therapies such as tyrosine kinase inhibitors that can be used to combat the molecular changes that promote cancer progression. Development of targeted therapies is not the only clinical benefit of gene analysis studies. Biomarkers identified from gene analysis can be used for early lung cancer detection, determine patient’s prognosis and response to therapy, and monitor disease progression. Biomarkers can be used to identify the NSCLC patient population that would most benefit from treatment (targeted therapies or chemotherapies), providing clinicians tools that can be used to develop a personalized treatment plan. This review explores the clinical potential of NSCLC genetic studies on diagnosing and treating NSCLC.

## Introduction

Lung cancer is the leading cause of cancer related deaths in both men (28 %) and women (27 %) in the United States [1]. In 2015, the American Cancer Society estimates 221,200 patients will be diagnosed with lung cancer and 158,040 deaths will occur due to this disease [1]. The majority of lung cancers are diagnosed at a late stage resulting in a 5 year survival rate (17 %) that is lower than breast (89 %), prostate (99 %), and colon carcinomas (65 %) [1]. The high death rates associated with lung cancer highlight the need for improved diagnosis and treatment procedures. Lung cancer is broken down into two main categories, non-small-cell lung cancer (NSCLC) and small-cell lung cancer (SCLC) based on cell morphology. NSCLC accounts for 85 % of all lung cancers and is subcategorized into pulmonary adenocarcinomas, squamous cell carcinomas, and large cell carcinomas (LCC) [2]. Forty, twenty-five, and ten percent of all lung cancers are diagnosed as adenocarcinomas, squamous cell carcinomas, and large cell carcinomas, respectively [2].

The first step to developing new treatments for any disease is to understand the molecular biology driving its progression. Development and implementation of high-throughput genomic technologies such as next generation sequencing (NGS) enable genotyping of tumors at a lower cost and quicker turn around than was previously possible [3, 4]. NGS and other nucleic acid sequencing technologies (reviewed in [3, 4]) have identified genetic mutations that drive lung cancer progression often referred to as “oncogenic drivers”. Discovery of lung cancer drivers has led to the development of therapies that target cancer cells and inhibit the pathways that promote lung cancer growth and progression. The use of targeted therapies have led to an increase in survival of lung cancer patients with certain genetic alterations such as epidermal growth factor receptor (EGFR) activating mutations and anaplastic lymphoma kinase (ALK) rearrangements [5–7]. Gefitinib, a small molecule inhibitor for the EGFR receptor was approved for use to treat NSCLC in May of 2003 [6]. Gefitinib was one of the first tyrosine kinase inhibitors and targeted therapies approved for NSCLC with aberrant activation of the EGFR pathway [8]. Gefitinib is an example of how discoveries made by basic science researchers on molecular changes in cancers can translate to provide therapies that can be utilized in the clinic.

However, the clinical significance of studying lung cancer mutations goes beyond drug development. Investigation and characterization of NSCLC mutations can also be used to identify biomarkers that can be used for early diagnosis and predicting patients’ prognosis and response to treatment that can be used to determine a personalized therapeutic regimen. This review will explore the clinical benefits of genetic mutation investigations for patients with NSCLC with an emphasis on use of gene biomarkers for diagnosis and treatment.

## Review

### Lung cancer mutations and targeted therapy

#### Mutations in NSCLC

NSCLC is a heterogeneous disease marked with a high rate of somatic mutations. Genetic analyses of the NSCLC subtypes lung adenocarcinomas and squamous cell carcinomas found a higher rate of mutations in these cancers than acute myelogenous leukemia, glioblastoma multiforme, and cancers of the breast, ovaries, and colon [9, 10]. The Cancer Genome Atlas (TCGA) research network and other groups, have identified several genes altered (by mutations, amplification, or rearrangements) in adenocarcinomas such as: EGFR, EML4-ALK (Echinoderm microtubule associated protein-like protein 4 fused with Anaplastic Lymphoma Kinase), KRAS (Kirsten rat sarcoma viral oncogene homolog), MET (mesenchymal-epithelial transition factor), ROS1 (c-ros oncogene 1), RET (rearranged during transfection), BRAF (v-Raf murine sarcoma viral oncogene homolog B1), and TP53 (tumor suppressor protein 53) and in squamous cell carcinomas: FGFR1 (fibroblast growth factor receptor 1), PIK3CA (phosphoinositide-3-kinase catalytic subunit alpha isoform), DDR2 (discoidin domain receptor 2), MET, SOX2 (SRY related HMG box gene 2), PTEN (phosphatase and tensin homolog), CDKN2A (TP53 and cyclin-dependent kinase inhibitor 2A) highlighted in Table 1 [9, 10].

**Table 1:** Lung adenocarcinoma and squamous cell carcinoma mutations, incidence, downstream effects, and targeted therapies

Genetic alterations	Incidence (%)	Downstream effect	Targeted therapy^a^	Sources
Adenocarcinomas				
EGFR (mut)	~15	↑ Proliferation, survival, angiogenesis, and metastasis	*Gefitinib*, *erlotinib*, *afatinib*, AZD9291, AZD8931	[10, 11]
EML4-ALK (fus)	2–7	↑Proliferation, survival, and migration	*Crizotinib*, *ceritinib*, alectinib	[11, 79, 80]
KRAS (mut)	~30	↑ Chemoresistance, proliferation, and survival	N/A	[10]
MET (amp)	3–5	↑ Cell survival, proliferation, and metastasis	Tivantinib, crizotinib cabozantinib, ornatuzumab	[11, 13, 24, 81]
ROS1 (fus)	1–2	↑ Survival	Foretinib & crizotinib	[13, 24]
RET (fus)	1–2	↑Proliferation	Carbozantinib, vandetanib, ponatinib	[11]
BRAF (mut)	5–10	↑ Resistance to EGFR inhibitors, proliferation, and survival	Debrafenib, sorafenib	[10, 24]
TP53 (mut)	46	↑ Growth, ↓ apoptosis	N/A	[10, 11]
Squamous cell carcinomas				
FGFR1 (amp)	16–25	↑ Proliferation, survival, and chemoresistance; ↓ patient prognosis	Nintendanib, ponatinib, AZD4547, dovitinib	[9, 11, 12, 82]
PIK3CA (mut)	8–18	↑ Proliferation and survival	Buparlisib, PX-866, BYL719, GDC-0941 or inhibitors for AKT: AZD5363, MK-2206	[9, 11, 13, 24, 82]
DDR2 (mut)	4	↑ Cell migration, invasion, proliferation, and survival	Dasatinib	[9, 13]
MET (amp)	3	↑ Cell survival, proliferation, and metastasis	Tivantinib, crizotinib cabozantinib, onartuzumab	[12, 24, 81]
SOX2	21	↑ Proliferation	N/A	[9]
PTEN (mut & del)	15–29	↑PI3K signaling, proliferation, and survival	PI3K inhibitors: buparlisib, PX-866, BYL719, GDC-0941 or inhibitors for AKT: AZD5363, MK-2206	[9, 11, 24, 82]
TP53 (mut)	81	↑ Growth, ↓ apoptosis	N/A	[9, 11, 12]
CDKN2A (del)	51	↑ Growth	N/A	[9]

*N*/*A* not available, currently not able to target efficiently with drugs, *Mut* mutations, *Del* deletions, *Amp* amplifications, *Fus* fusions

^a^For detailed review of targeted drugs and clinical trials see [2, 11, 13, 24]

A large majority of altered genes code for proteins that are involved in receptor tyrosine kinase (RTK) signaling, leading to the promotion of NSCLC cell proliferation, survival, migration, and invasion (Table 1) [9–13]. Several mutations or amplifications occur in genes for the RTKs: EGFR, ALK, MET, ROS1, RET, FGFR1 and DDR2 or in genes that help facilitate RTK downstream signaling events such as: KRAS, BRAF, and PIK3CA (phosphoinositide-3-kinase (PI3K) family member) in NSCLC [9–13]. PTEN mutations and/or deletions occur in 15–20 % of squamous cell carcinomas and leads to aberrant activation of the PI3K pathway (Table 1). Although genetic alterations occur in both adenocarcinomas and squamous cell carcinomas effecting RTK signaling, these NSCLC sub-types have different dominant mutations, amplifications, or rearrangements (Table 1). For example, EGFR and KRAS are two of the most commonly mutated genes in lung adenocarcinomas; however they are rarely mutated in lung squamous cell carcinomas (Table 1) [9, 10].

The advent of technology and assays that allow the sequencing and analysis of the genome has resulted in an influx of information regarding NSCLC and increased the number of possible therapeutic targets. The NSCL genetic alterations represented in Table 1 barely scratches the surface in regards to the many different genomic abnormalities that have been identified. The question remains which of the identified mutations constitute good targets for therapeutic intervention in NSCLC? However, these genetic alterations are of interest to researchers for several reasons including; the rate of occurrence in NSCLC and/or the ability of these changes in the genome to drive oncogenesis. The rate of occurrence in NSCLC and the ability of these changes in the genome to drive oncogenesis are two criteria used to determine pharmaceutical targets [14]. In the next section, we will highlight the targeted therapies that are currently approved or in development for NSCLC.

#### Targeted therapies for NSCLC

Identification of gene mutations, amplifications, and rearrangements in NSCLC provides a pool of new targets to be used in drug development. Targeted therapies include small molecular inhibitors and monoclonal antibodies that bind and inhibit the molecular pathways that promote NSCLC progression. Introduction of targeted therapies to the clinic resulted in decreased toxicity and increased rate of disease-free survival for patients with NSCLC compared to those treated with standard chemotherapy [15–18]. Unfortunately, only a small percentage of patients with NSCLC are eligible for approved targeted therapies. Patients eligible for FDA approved targeted therapies (erlotinib, afatinib, crizotinib, and ceritinib) are those diagnosed with advanced stage lung adenocarcinomas with activating mutations in the EGFR gene or EML4-ALK fusions [2, 13, 19–22]. Lung squamous cell carcinomas and adenocarcinomas have differing genetic profiles and molecular drivers and therefore do not respond to the tyrosine kinase inhibitors approved for treatment of NSCLC [23].

Several reviews have been published this year on NSCLC targeted therapies; therefore this review contains a short description of targeted therapies approved or in development for NSCLC. For more information regarding targeted therapies in development and ongoing clinical trials see the following reviews [2, 11, 13, 24]. Table 1 lists targeted therapies that are approved (bold font) or undergoing clinical trials for NSCLC and the effected pathways. Crizotinib is a tyrosine kinase inhibitor that inhibits ALK and other RTKs such as MET and ROS1 [25]. Crizotinib is currently approved for treatment of NSCLC that contain ALK rearrangements and is undergoing clinical trials for NSCLC with ROS1 rearrangements [26, 27]. Several compounds are undergoing clinical trials for targeted treatment of lung adenocarcinomas and lung squamous cell carcinomas with genetic alterations in MET, BRAF, ROS1, RET, FGFR1, and PIK3CA (Table 1). Examples of ongoing clinical trials can be seen in Table 2. Not all of the identified genetic abnormalities in NSCLC (KRAS, SOX2, CDKN2A, and TP53) are currently targetable by small molecule inhibitors or monoclonal antibodies [11, 13, 28]. However, therapies that target downstream signaling pathways for KRAS such as MEK (mitogen-activated protein kinase kinase 1) inhibitors are being investigated [24].

**Table 2:** Examples of targeted therapies currently undergoing clinical testing in NSCLC

Study name/identifier^a^	Purpose	Targeted therapies
Pilot trial of molecular profiling and targeted therapy for advanced NSCLC, SCLC, and thymic malignancies *NCT01306045*	Evaluate the ability of genetic analysis to determine targeted therapy for patients with NSCLC	AZD6244 MEK1 inhibitor for KRAS or BRAF mutations
		MK-2206 AKT inhibitor for PIK3CA, AKT, PTEN mutations
		Sunitib inhibitor for KIT or PDGFRA mutations
		Lapatinib inhibitor for ERBB2
		Erlotinib inhibitor for EGFR mutations
Evaluation of the efficacy of high throughput genome analysis as a therapeutic decision tool for patients with metastatic NSCLC *NCT02117167*	Evaluate the use of high throughput genome analysis to determine administration of targeted therapy according to genetic profile of the tumor compared to standard treatment. Randomized multicenter Phase II trial	AZD2014 inhibitor for m-TOR
		AZD4547 inhibitor for FGFR
		AZD5363 inhibitor for AKT,
		AZD8931 inhibitor for EGFR
		Selumetinib inhibitor for MEK1
		Vandetanib inhibitor for VEGFR and EGFR
FGFR1 amplification as a predictor of efficacy in a biomarker-driven phase II study of BIBF 1120 in advanced lung SCC patients who have failed up to 2 prior chemotherapeutic regimens *NCT01948141*	Evaluate efficacy of nintedanib in treating patients with advanced NSCLC who failed up to two previous chemotherapy regimens and use of FGFR1 gene copy number is a predictive biomarker	Nintedanib TKI for FGFR1

*AKT* v-akt murine thymoma vial oncogene homolog 1, *BRAF* v-raf murine sarcoma viral oncogene homolog B, *EGFR* epidermal growth factor receptor, *FGFR1* fibroblast growth factor receptor 1, *KRAS* v-Ki-ras2 Kirsten rat sarcoma viral oncogene homolog, *MEK* mitogen-activated protein kinase kinase 1, *m-TOR* mechanistic target of rapamycin, *NSCLC* non-small cell lung carcinoma, *SCC* squamous cell carcinoma, *SCLC* small cell lung carcinoma, *VEGF* vascular endothelial growth factor, *VEGFR* vascular endothelial growth factor receptor

^a^Denotes http://www.clinicaltrials.gov identifier

One of the largest problems associated targeted therapies is primary or acquired resistance. Primary resistance to EGFR inhibitors have been found in patients that harbor KRAS or certain EGFR point mutations (T790) [29–31]. However, mutations of KRAS and EGFR are mutually exclusive [10]. Expression of Bim after treatment with tyrosine kinase inhibitors is also associated with primary resistance. Patients who have low expression of the pro-apoptotic protein Bim or have polymorphisms altering the function of Bim have lower response to EGFR and ALK inhibitors [31]. Use of tyrosine kinase inhibitors gefitinib, erlotinib, and crizotinib for NSCLC treatment invariably leads to acquired drug resistance [32, 33]. Mutations in the EGFR gene (T790M) or alterations in MET(amplification), BRAF(mutation), HER2 (amplification), PIK3CA (mutation), or PTEN (loss) leads to gefitinib and erlotinib resistance [33]. Resistance to ALK inhibitors occurs due to mutations in ALK (L1196M), amplifications of ALK, or activation of EGFR or KRAS that bypass the inhibition induced by tyrosine kinase inhibitor crizotinib [19, 34, 35]. Second generation inhibitors afatinib and ceritinib are approved for use in the FDA and have shown some promise in patients who were previously treated with first generation tyrosine kinase inhibitors for EGFR or ALK (respectively) [19, 34, 36]. In a phase IIb/III trial, afatinib increased progression free survival (3.3 months) of patients with advanced NSCLC that progressed after treatment with erlotinib, gefitinib, or both compared to placebo group (1.1 months P < 0.0001) [36]. In a phase I trial, ceritinib in 114 patients with advanced NSCLC had an overall response rate of 58 % [19, 34]. Response rate for ceritinib in patients who had disease progression after use of crizotinib was 56 %. Genetic analysis of 19 tumors from patients that previously had crizotinib therapy, showed patients responded to ceritinib even if they had the presence of ALK resistance mutation (L1196M) [19, 34].

Targeted therapeutics is a growing field in which there is a great need for therapies (1) that can be used for squamous cell carcinoma patients and (2) that overcome resistance associated with the available targeted therapies. Targeted therapies give the clinician the ability to select NSCLC treatments based on the molecular characteristics of the patients’ cancer. Personalized therapy addresses the fact that cancers are a heterogeneous disease in which no two tumors are identical. The generalized therapeutic approach commonly used does not take into account molecular mechanisms that lead to chemoresistance and recurrence [37]. Clinical validation is needed to determine if genetic analysis based selection of targeted therapies can increase patient survival. Several clinical studies are evaluating the use of genetic analysis to determine a personalized treatment plan for NSCLC patients using several different arms of targeted treatments. Two examples of ongoing clinical trials can be found in Table 2 in which researchers are using therapies that target pathways modulated in NSCLC. Choosing the optimal treatment is a difficult process, but the implementation of biomarkers can provide clinicians with information that can be used in selection of NSCLC treatment.

### Biomarkers and NSCLC

Seventy-nine percent of patients diagnosed with lung cancer present with regional or distant metastases [1]. The 5 years survival rate for patients diagnosed with early stage lung cancer is 27 and 50 % greater than patients diagnosed with regional or distant metastases, respectively [1]. The National Lung Screening Trial found that early detection of lung cancer using low-dose computed tomography (CT) resulted in a 20 % reduction in mortality [38]. However, CT scans are unable to differentiate between benign and malignant lesions. Follow-up lung biopsies found that 96.4 % of the lesions found by CT were benign [38]. The high percentage of false positive results, costs of CT scans, and stress to patients suggests CT scans are not practical for early lung cancer screening. Although early diagnosis gives an advantage toward patient survival, 36 % of patients with stage I to II NSCLC recur 5 years after surgery [39]. Gene mutations and protein expression can impact patient survival. For example, patients with deletions in exon 19 and mutations in exon 21 had a longer overall survival after treatment with gefitinib or erlotinib [7, 40]. High expression of ERCC1 (excision repair cross-complementation group 1) mRNA or protein is associated with decreased overall survival in NSCLC patients treated with platinum based therapies [41, 42].

Investigation of genetic alterations has enabled the identification of key molecular changes that occur during the pathogenesis of lung cancer that can be used as biomarkers for detecting and treating lung cancer. Biomarkers are detectable changes in DNA, RNA, protein, lipids, etc., that can be used for (1) detecting and diagnosing lung cancer, (2) determining prognosis, (3) predicting patient response to therapy, and 4) choosing an optimal treatment regimen for NSCLC patients [43–45]. Utilization of biomarkers will allow clinical oncologists to select a personalized treatment option that will give the patient the greatest chance of survival, decrease toxicity, and unnecessary procedures [44, 45]. Investigative studies for lung cancer biomarkers have been conducted using cancer tissue, blood, lung condensate, sputum, and saliva samples [46–51]. In the following sections, we will explore the clinical potential and benefits of using gene biomarkers in the clinic to detect and treat NSCLC.

#### Gene biomarkers: implications for early detection and diagnosis of NSCLC

Biomarkers extracted from biofluids (blood, sputum, and saliva) are under investigation for the diagnosis of lung cancer without the use of invasive procedures required to collect lung cancer or bronchial epithelial cells [52, 53]. Blood samples extracted from NSCLC patients contain circulating cell free DNA (cfDNA; normal and tumor DNA), RNA, and mononuclear and tumor cells all of which are sources of genetic material that can be used for identification of biomarkers to detect lung cancer [52, 54, 55]. A multi-marker panel consisting of the circulating expression of cfDNA, mRNA expression of peptidylarginine deaminase type 4 (PADA) and proplatelet basic protein (PPBP) was able to discriminate between patients with NSCLC and controls with a sensitivity of 92 % and a specificity of 89 % [56]. Increased expression of cfDNA can be detected in the peripheral blood of NSCLC patients as early as stage IA compared to controls demonstrating the potential of cfDNA to be used in early detection of lung cancer [57]. Although cfDNA can be detected as early as stage I, it is associated with a lower sensitivity. Using a new strategy to analyze circulating tumor cells known as cancer personalized profiling by deep sequencing (CAPP-Seq), Newman et al. were able to detect circulating tumor DNA in 100 % of NSCLC between the stages of II–IV and 50 % of stage I tumors with specificity of 96 % for both groups [58]. Studies in peripheral blood mononuclear cells have examined gene expression profiles in order to identify biomarkers that can be used for diagnosis of breast, urinary bladder, and lung cancers [54, 59, 60]. Showe et al. [54] identified a 29 mRNA signature from peripheral mononuclear cells of patients with NSCLC that distinguished with 91 % sensitivity and 80 % specificity patients with cancer from controls. Clinical trials are vital to validate biomarkers before use in the clinic. Search of http://www.clinicaltrials.gov yielded only one clinical trial based on our search parameters that is looking for gene biomarkers in the blood that can be used for diagnosis of NSCLC (http://www.clinicaltrials.gov identifier NCT02169349; Table 3).

**Table 3:** Clinical studies for biomarkers under investigation for diagnosis of NSCLC

Study name	Identifier^a^	Purpose	Biomarker(s)
Detection and diagnosis of NSCLC			
Prospective blinded evaluation of salivary transcriptome biomarkers for NSCLC detection	NCT02294578	Evaluate sensitivity and specificity of biomarkers (mRNA) from saliva of patients with lung lesions prior to cancer diagnosis	BRAF, CCNI, EGFR, FGF19, FRS2, GREB1, and LZTS1
Molecular Diagnosis on circulating tumor DNA of NSCLC (ANTiCIPe)	NCT02169349	Develop an assay to detect DNA mutation in circulating tumor DNA (from plasma) for diagnosis, treatment, and monitoring cancer progression of NSCLC (stage IIIb and IV)	N/S

*N*/*S* biomarkers were not specified, *BRAF* v-raf murine sarcoma viral oncogene homolog B1, *CCN1* cycline I, *EGFR* epidermal growth factor receptor, *FGF19* fibroblast growth factor 19, *FRS2* fibroblast growth factor receptor substrate 2, *GREB1* growth regulation by estrogen in breast cancer 1, *LZTS1* leucine zipper, putative tumor suppressor 1

^a^Denotes http://www.clinicaltrials.gov identifier

Gene expression analyses of saliva and sputum samples have identified possible biomarkers that have clinical potential in detecting NSCLC. In 2012 Zhang et al. [46] identified and pre-validated seven mRNA transcripts [BRAF (v-raf murine sarcoma viral oncogene homolog B1), CCNI (cyclin I) EGFR, FGF19 (fibroblast growth factor 19), FRS2 (fibroblast growth factor receptor substrate 2), GREB1 (growth regulation by estrogen in breast cancer 1), and LZTS1 (leucine zipper, putative tumor suppressor 1)] expressed in the saliva from patients with NSCLC. A multi-marker panel measuring mRNA expression of CCN1, EGFR, FGF19, FRS2, and GREB1 in saliva, differentiated patients with lung cancer from controls with a sensitivity of 93.75 % and specificity of 82.8 % [46]. Currently, a clinical validation study evaluating the detection of lung cancer using a single or multi-marker panel of the seven mRNA transcripts identified by Zhang et al. [46] is recruiting patients with lung lesions suspected of having lung cancer (NCT02294578; Table 3).

Gene alterations have been identified from sputum samples collected from NSCLC patients suggesting sputum can be used to identify genetic abnormalities in lung cancer [51, 61, 62]. Gene analysis performed on the sputum of 49 NSCLC patients, 49 patients with COPD, and 49 healthy smokers, identified 15 genes with significant differences in copy number in patients with NSCLC compared to controls. A multi-marker panel containing 6 of the identified altered genes (ENO1, FHIT, HYAL2, SKP2, CDKN2A, and 14-3-3zeta) were able to differentiate between patients with NSCLC and control with 86.7 % specificity and 93.9 % sensitivity [63]. Further studies are needed to validate biomarkers in the sputum prior to use in the clinic. The ability to detect the presence of NSCLC from fluid samples can aid in decreasing the cost and stress to patients associated with current methods (biopsies and CT scans) used to differentiate between a benign and malignant small nodule that detected using CT scans. However, more sensitive methods are needed to detect the presence of stage I lung cancer.

#### Gene biomarkers: implications for use as NSCLC predictive and prognostic biomarkers

Several studies have examined the genetic profiles of NSCLC to identify prognostic and predictive gene biomarkers. A prognostic biomarker is a measureable factor that can be used to ascertain the likelihood of an event such as; recurrence, drug resistance, or metastasis to occur irrespective of treatment [64]. Whereas predictive biomarkers provide information concerning the benefits of a treatment for the patient such as increased survival or tumor response [64]. Prognostic and predictive biomarkers can be used to tailor chemotherapy treatments based on gene mutations or expression resulting in increased patient survival. Several studies have investigated the efficacy of selecting chemotherapies based on expression of genes ERCC1 (excision repair cross complementing gene 1), RRM1 (ribonucleotide reductase), TS (thymidylate synthase), TUBB3 (type III β-tubulin), and BRCA1 (breast cancer susceptibility gene 1) that are associated with chemoresistance in NSCLC [42, 47, 48, 65]. Miao et al. [48] conducted a retrospective analysis and found that chemotherapy selection based on gene expression of ERCC1 (platinum based therapies resistance), RRM1 (gemcitabine resistance), TS (pemetrexed resistance), and TUBB3 (vincristine resistance) in tumors resulted in higher rate of disease free survival [1 year = 66.7 vs. 44.7 % (P = 0.014); 2 year 48.9 vs. 27.2 % (P = 0.010)] in stage IIIA NSCLC patients [48]. Furthermore, Zhang et al. [65] found that patients given a tailored chemotherapy regimen based on presence of ERCC1, RRM1, and TUBB3 had higher 1 year survival rates (69.5 %) and longer progression free survival time (5.2 months) compared to patients given standard treatment of gemcitabine plus cisplatin [survival rate = 40.9 % (P = 0.021) and progression free survival time 4.1 months (P = 0.0260)]. The results from these studies suggest personalized therapy selection based on expression of genes associated with chemoresistance can be used to increase patient response. However, these studies did not find a statistically significant change in overall survival and more validation needs to be done before implementation in the clinic. Clinical validation studies NCT01424709, NCT00792701, and NCT02145078 are investigating the effect of selecting chemotherapy for NSCLC patients using expression of ERCC1, TS, RRM1, and BRCA1, mRNA in different combinations, on patient response and survival (Table 4).

**Table 4:** Clinical studies for biomarkers under investigation for predicting response or determining prognosis of patient with NSCLC to treatment

Study name	Identifier^a^	Purpose	Biomarker(s)
The identification, validation, and implementation of prognostic and/or predictive biomarkers for adjuvant chemotherapy in early stage NSCLC	NCT01595074	Evaluate biomarkers for predicting treatment response in samples from patients with early-stage NSCLC (IA–IIIA) previously treated with adjuvant chemotherapy	15 Gene prognostic and predictive mRNA signature
Adjuvant lung cancer enrichment marker identification and sequencing (ALCHEMIST)	NCT02194738	Evaluate the use of genetic testing to screen patients with stage IB–IIIA NSCLC that will or have undergone surgery.	EGFR, PIK3CA, PTEN, ALK, & other N/S
A study to assess the ability to initiate therapy in advanced NSCLC patients based on genetic analyses of tumor specimens	NCT02178163	Evaluate use of genomic analysis of tissue samples from patients with reoccurred or stage IV NSCLC to select therapy treatment	N/S
A study of predictive and prognostic markers in patients with NSCLC	NCT00958555	Investigate predictive and prognostic markers from circulating tumor cells in blood and tumor for outcome after treatment for NSCLC	Genes linked to genetic risk of NSCLC (N/S)
A pilot study to evaluate the predictive value of circulating tumor DNA for clinical outcome in patients with advanced head and neck and lung cancers	NCT02245100	Evaluate predictive value of DNA from circulating tumor cells in patients with stage III–IV NSCLC to treatment	N/S
A prospective study of plasma genotyping as a noninvasive biomarker for genotype-directed cancer care	NCT02279004	Evaluate genotyping oncogenic mutations using blood samples from NSCLC patients to determine cancer genotype and patient response to therapy	EGFR, KRAS, BRAF
Prognosis or predicting response			
Individualized 1st line chemotherapy based on BRCA1 and RRMI mRNA expression levels for advanced NSCLC	NCT01424709	Compare survival rate of patients with advanced NSCLC based on gene expression compared to patients given gemcitabine/cisplatin	RRM1 and BRCA1 mRNA
Phase II ERCC1 and RRMI1-based adjuvant therapy trial in patients with stage I NSCLC	NCT00792701	Compare survival of stage I NSCLC patients that have undergone surgery given chemotherapy based on gene expression compared to patients given gemcitabine/cisplatin	ERCC1 & RRM1
Pilot trial of docetaxel, gemcitabine, or pemetrexed single agent therapy with serial tumor specimen collection in patients with advanced NSCLC	NCT02145078	Evaluate if levels of certain genes in tissue and blood samples are related to patient’s response to chemotherapy (stage IV NSCLC) and compare markers between CTCs, PBMCs, and tumor	RRM1, TS, BRCA1 & other molecules N/S

*N*/*S* biomarkers were not specified, *BRAF* v-raf murine sarcoma viral oncogene homolog B1, *BRCA1* breast cancer susceptibility gene 1, *CTC* circulating tumor cells, *EGFR* epidermal growth factor receptor, *ERCC1* excision repair cross complementing gene 1, *KRAS* v-Ki-ras2 Kirsten rat sarcoma viral oncogene homolog, *PBMC* peripheral blood mononuclear cells, *PTEN* phosphatase and tensin homolog, *RRM1* ribonucleotide reductase M1, *TS* thymidylate synthase, *NSCLC* non-small cell lung cancer

^a^Denotes http://www.clinicaltrials.gov identifier

The implementation of targeted therapies has resulted in a need for predictive biomarkers to determine patient population that would benefit from their use. EGFR mutations and ALK rearrangements are examples of predictive biomarkers that are tested prior to use of gefitinib, erlotinib, afatinib, crizotinib, and ceritinib [7, 66, 67]. In 2013, the College of American Pathologists, International Association for the Study of Lung Cancer, and Association for molecular Pathology established guidelines for the molecular testing of all advanced stage lung adenocarcinomas for EGFR mutations and ALK rearrangements prior to treatment [68]. Several clinical trials are being conducted to test the efficacy of new targeted therapies in conjunction with evaluating predictive biomarkers for the treatment of NSCLC. An example of this can be found in Table 2 in which the gene copy number of FGFR1 is being tested to predict patient response to the tyrosine kinase inhibitor nintedanib undergoing clinical evaluation for treatment of NSCLC with FGFR1 amplifications (Table 2).

Molecular profiling of NSCLC is generally performed on biopsies that contain a small amount of tumor and stromal cells and are extracted by using invasive bronchoscopy or transthoracic needle biopsy. Therefore biomarker analysis using tumor tissue for selection of treatment for patients must be highly sensitive, selective, cost effective, reproducible, and have a quick turn-around time [4, 44, 69]. Furthermore, different areas of a tumor and metastatic sites can have dissimilar genetic profiles making it difficult to identify and extract biomarkers to monitor disease progression and response to therapy [4, 44, 69]. Peripheral blood samples containing circulating tumor cells (CTC) and free circulating tumor DNA (ctDNA) may provide a source to measure biomarkers prior to treatment. EGFR activating mutations associated with response to TKIs have been identified in DNA extracted from CTCs and in free ctDNA [70–73]. EGFR mutations analyzed from peripheral blood samples correlated with DNA analysis from matched tumor tissue; however ctDNA had a greater sensitivity for detecting EGFR mutations than DNA extracted from CTCs [70, 72, 73]. An analysis conducted by Yung et al. [73] was able to identify patients with EGFR mutations using ctDNA from plasma with an 92 % sensitivity and 100 % specificity compared to analysis using DNA extracted from NSCLC patients. Mutations in KRAS and ALK rearrangements have also been found in analyses on CTCs or ctDNA and correlate with mutation analysis in match tumor samples [74, 75]. A list representing ongoing clinical trials investigating the use of biomarkers as predictive and prognostic factors from tumors and peripheral blood, can be found in Table 4.

Biomarkers extracted from blood, saliva, and sputum have the possible advantage of providing early diagnosis of lung cancer and information that can be used for treatment selection without the use of invasive procedures. Multiple specimens can also be analyzed allowing the capabilities to monitor disease progression and response to therapy. This review highlighted several biomarker studies for use detecting and treating NSCLC. However, validation studies and standardization of biomarker analysis are needed before biomarkers are employed in the clinic [76].

## Conclusions

Analysis of gene alterations has provided invaluable insight that has spurred the development of targeted therapies for NSCLC. The benefits of understanding the molecular changes promoting lung carcinogenesis go beyond drug development. Identification and validation of biomarkers that can be used to provide early detection and aid in the selection of personalized therapy are currently undergoing clinical testing. Biomarkers for early detection of lung cancer have the potential to be combined with current CT scans or alone to identify the presence of cancers without requiring invasive procedures. Utilization of biomarkers in the clinic for determination of patients’ prognosis and response to therapy are invaluable tools that may increase patient survival and decrease toxicity due to ineffective treatments. However, understanding the genetic changes in NSCLC is just one side of the coin. Transcriptomic and proteomic studies not covered in this review are vital in understanding the downstream effect of gene mutations; and in identifying other mechanisms that promote cancer progression. DNA methylation, miRNA, and protein expression are all possible types of biomarkers that can be used for early detection, determining prognosis, and predicting patient response to therapy in NSCLC [43, 77, 78].

## Abbreviations

AKT: v-akt murine thymoma vial oncogene homolog 1
ALK: Anaplastic lymphoma kinase
BRAF: v-raf murine sarcoma viral oncogene homolog B
CT: Computed tomography
cDNA: Circulating DNA
ctDNA: Circulating tumor DNA
CTC: Circulating tumor cells
EGFR: Epidermal growth factor receptor
EML4-ALK: Echinoderm microtubule associated protein like 4 and anaplastic lymphoma kinase fusion
FGFR1: Fibroblast growth factor receptor 1
KRAS: v-Ki-ras2 Kirsten rat sarcoma viral oncogene homolog
NSCLC: Non-small cell lung cancer
SCLC: Small-cell lung cancer
